# Complement-Opsonized HIV-1 Overcomes Restriction in Dendritic Cells

**DOI:** 10.1371/journal.ppat.1005005

**Published:** 2015-06-29

**Authors:** Wilfried Posch, Marion Steger, Ulla Knackmuss, Michael Blatzer, Hanna-Mari Baldauf, Wolfgang Doppler, Tommy E. White, Paul Hörtnagl, Felipe Diaz-Griffero, Cornelia Lass-Flörl, Hubert Hackl, Arnaud Moris, Oliver T. Keppler, Doris Wilflingseder

**Affiliations:** 1 Division of Hygiene and Medical Microbiology, Medical University of Innsbruck, Innsbruck, Austria; 2 Institute of Medical Virology, University of Frankfurt, Frankfurt, Germany; 3 Division of Medical Biochemistry, Biocenter, Medical University of Innsbruck, Innsbruck, Austria; 4 Department of Microbiology and Immunology, Albert Einstein College of Medicine, Bronx, New York, New York, United States of America; 5 Central Institute for Blood Transfusion & Immunological Department, Medical University of Innsbruck, Innsbruck, Austria; 6 Division of Bioinformatics, Biocenter, Medical University of Innsbruck, Innsbruck, Austria; 7 Sorbonne Universités, Université Pierre et Marie Curie (UPMC)-Paris 6, Center for Immunology and Microbial Infections - Centre d’Immunologie et des Maladies Infectieuses (CIMI-Paris), Paris, France; 8 INSERM, U1135, Center for Immunology and Microbial Infections - Centre d’Immunologie et des Maladies Infectieuses (CIMI-Paris), Paris, France; 9 CNRS, ERL 8255, Center for Immunology and Microbial Infections - CIMI-Paris, Paris, France; 10 Assistance Publique – Hôpitaux de Paris (AP-HP), Hôpital Pitié-Salpêtrière, Department of Immunology, Paris, France; University of Pennsylvania School of Medicine, UNITED STATES

## Abstract

DCs express intrinsic cellular defense mechanisms to specifically inhibit HIV-1 replication. Thus, DCs are productively infected only at very low levels with HIV-1, and this non-permissiveness of DCs is suggested to go along with viral evasion. We now illustrate that complement-opsonized HIV-1 (HIV-C) efficiently bypasses SAMHD1 restriction and productively infects DCs including BDCA-1 DCs. Efficient DC infection by HIV-C was also observed using single-cycle HIV-C, and correlated with a remarkable elevated SAMHD1 T592 phosphorylation but not SAMHD1 degradation. If SAMHD1 phosphorylation was blocked using a CDK2-inhibitor HIV-C-induced DC infection was also significantly abrogated. Additionally, we found a higher maturation and co-stimulatory potential, aberrant type I interferon expression and signaling as well as a stronger induction of cellular immune responses in HIV-C-treated DCs. Collectively, our data highlight a novel protective mechanism mediated by complement opsonization of HIV to effectively promote DC immune functions, which might be in the future exploited to tackle HIV infection.

## Author Summary

We here give insight into a substantial novel way of dendritic cell modulation at least during acute HIV-1 infection by triggering integrin receptor signaling. We found that complement-opsonization of the virus is able to relieve SAMHD1 restriction in DCs, thereby initiating strong maturation and co-stimulatory capacity of the cells and stimulating efficient cellular and humoral antiviral immune responses. This newly described way of DC modulation by complement might be exploited to find novel therapeutic targets promoting DC immune functions against HIV.

## Introduction

Dendritic cells (DCs) are key regulators of immunity given their pivotal role in initiating and shaping adaptive immune responses against a vast array of pathogens and cancers [1–3]. HIV-1 has evolved strategies to evade DC-mediated antiviral immunity, i.e. inefficient replication. When restriction to HIV-1 replication in DCs was abrogated by simian Vpx, DCs exerted a potent type I IFN response and co-stimulatory function [4]. Besides hiding from DC-mediated immunity by low-level infection, the virus additionally exploits DCs as shuttles to promote its own dissemination [5]. Rapid immune responses against pathogens are provided via DC-expressed pattern recognition receptors or complement receptors (CRs). The complement (C) system constitutes a first line of defense against HIV-1 at mucosal surfaces and the HIV-1 envelope expresses a C-activating domain [6–8]. Thus, the virus is spontaneously surrounded by covalently linked C-fragments and opsonized HIV-1 particles accumulate already during the acute phase of infection [6, 7]. These structures interact with the abundant CR3 and CR4 on DCs and not via DC-SIGN/gp120 as shown earlier by our group [9]. Complement-opsonization was found to play a decisive role in priming humoral responses as well as antiviral T cell immunity during different viral infections [10–14]. As shown by Manel et al. [4], Vpx-mediated ease of DC restriction [15] to HIV-1 replication allowed reverse transcription of HIV to proceed, thereby giving rise to type I IFN production, maturation of the cells and improved antigen presentation [4, 16]. Thus, enhanced DC infection was associated with an increased quality and quantity of virus-specific immune responses [4, 16]. More recently, Laguette et al. [17] identified SAMHD1 as dendritic- and myeloid cell-specific HIV-1 restriction factor, which was counteracted, if the accessory protein Vpx encoded in the SIV or HIV-2 genome was incorporated into viral particles [17–19]. SAMHD1 restriction in DCs as well as in quiescent CD4^+^T cells was overcome and the cells infected if SAMHD1 was degraded by Vpx-mediated actions [16, 17, 19]. Yet, phosphorylation of SAMHD1 on residue T592 was shown to negatively regulate its HIV-1-restricting ability without reducing cellular dNTP levels [20–23].

We here illustrate that C-opsonized HIV-1 (HIV-C) efficiently infects immature DCs (iDCs) to significantly higher levels compared to non-opsonized HIV (HIV). Overcoming HIV-1 restriction in DCs by HIV-C was associated with a highly increased phosphorylation of SAMHD1 T592, but not SAMHD1 degradation. Blocking SAMHD1 phosphorylation in HIV-C-exposed DCs significantly reduced HIV-1 replication, thus highlighting the role of SAMHD1 phosphorylation for effective DC infection. After defeating restriction, HIV-C-DCs showed increased expression of maturation markers and co-stimulatory molecules, of type I IFN-associated genes and proteins as well as significantly improved stimulation of HIV-specific CD4^+^ and CD8^+^ T cell clones. Our data provide the first evidence that complement opsonization of HIV-1 activates highly functional HIV-specific cellular immunity as well as type I IFN responses due to overcoming restriction mechanisms. Thereby, we here give novel mechanistic insights, how complement opsonization in concert with DCs might contribute to the decline of viremia during the acute phase of infection and this could be exploited for yet not considered, future therapeutic targets against HIV-1.

## Results

### DCs are efficiently infected by HIV-C

**Fig 1:**
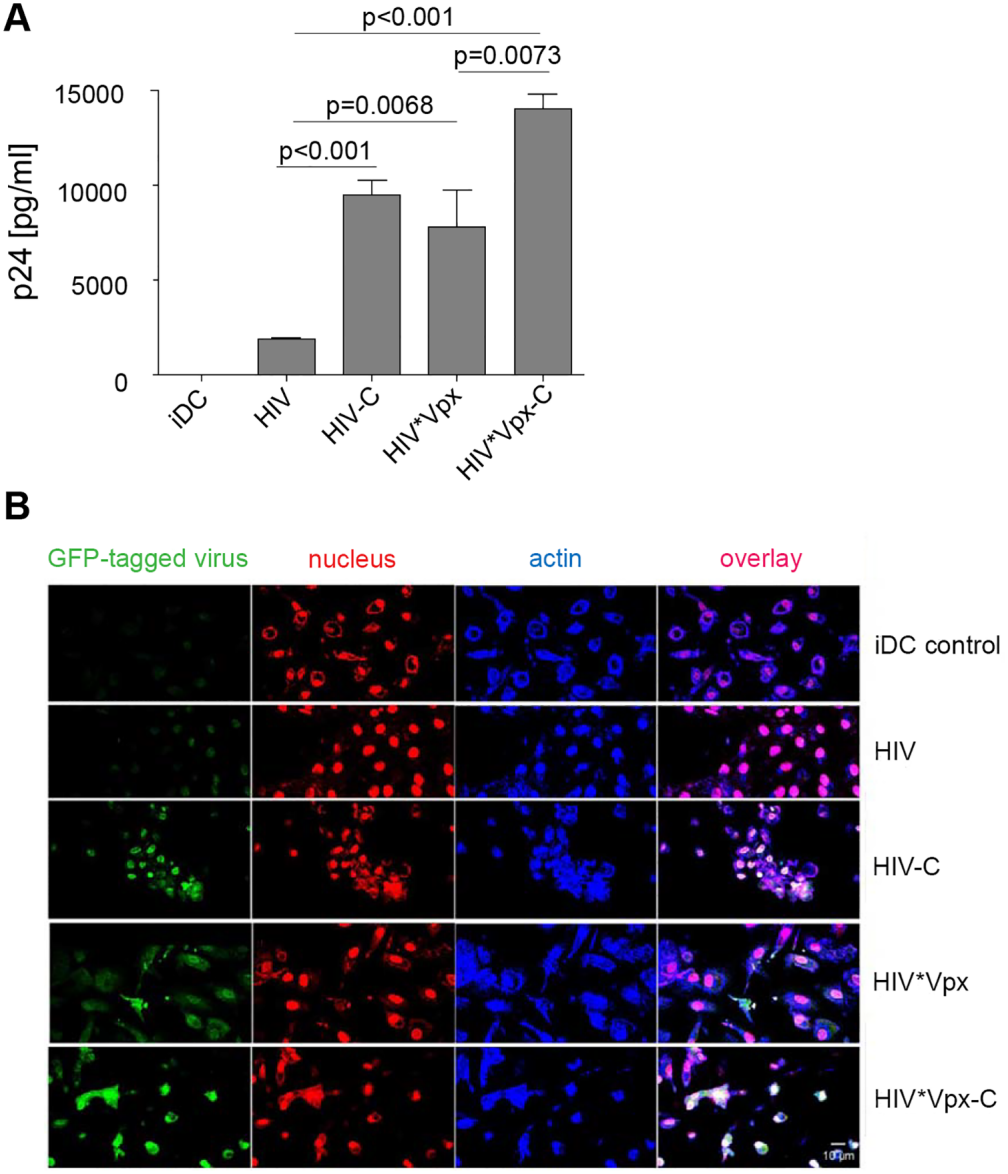
**(A) DCs are effectively infected with HIV-C**. Immature dendritic cells (iDCs) were infected with non-opsonized HIV (HIV*Vpx, HIV) and C-opsonized HIV (HIV*Vpx-C and HIV-C) DCs incubated with HIV show a significantly lower, moderate productive infection compared to HIV*Vpx (p = 0.0068), HIV*Vpx-C (p<0.0001) or HIV-C (p<0.0001) DCs on d11 p.i. Complement opsonization significantly enhanced the productive infection of DCs independent on Vpx incorporation (HIV-C, HIV*Vpx-C). Results of one (out of 5) representative infection assay performed in triplicates on d11 p.i. is depicted in Fig 1A. *(B) Complement opsonization of HIV increases infection of DCs independent on Vpx-incorporation*. Confocal microscopic analyses (original magnification 40xOIL) revealed that virus up-take in DCs using HIV*VpxGFP (4^th^ row) was enhanced when the virus was opsonized with C prior DC loading (HIV*VpxGFP-C, 5^th^ row). Also non-opsonized HIV (HIV) showed an increased up-take in DCs, when opsonized with complement (HIV-C, 3^rd^ row) compared to non-opsonized virus (HIV, 2^nd^ row). The virus is shown in green (GFP-tagged virus), the nucleus in red (nucleus) and actin staining in blue (Actin). Non-infected iDCs (iDCs control) served as negative controls (1^st^ row) and 40 cells were analyzed per condition. Scale: 10 μm

As demonstrated, efficient antiviral T cell responses are initiated, when DCs are productively infected by HIV-1 after their resistance to infection has been circumvented [4, 24]. In contrast, the inability of DCs to become infected is supposed to be a protective mechanism for HIV-1 survival. To characterize the effect of C-opsonization of HIV-1 on DCs, we infected DCs with non-opsonized HIV or HIV-C. To control productive DC infection, differentially opsonized Vpx-carrying HIV-1 preparations (HIV*Vpx, HIV*Vpx-C) were used [19]. As expected, viral replication in DCs exposed to control HIV*Vpx was significantly higher (p = 0.0068) than the low-level infection observed in HIV-DCs (Fig 1A). Complement-opsonized HIV depicted a 5-fold higher productive DC infection compared to non-opsonized HIV (Fig 1A and S1A Fig; mean p24 values of DCs for HIV 1870 pg p24/ml vs. HIV-C 9500 pg p24/ml). Remarkably, HIV-C caused a significantly higher productive infection (p<0.0001) in DCs compared to HIV and a slightly higher infection than that mediated by the positive control HIV*Vpx (Fig 1A). Opsonization of HIV*Vpx furthermore considerably enhanced the productive DC infection compared to HIV- (p<0.0001) or HIV*Vpx- (p = 0.0073) exposed DCs (Fig 1A). S1A Fig depicts the increase in p24 production by HIV-C, HIV*Vpx and HIV*Vpx-C, while non-opsonized HIV showed a low replication in DCs over time (S1A Fig). Increased infection of DCs by HIV-C was furthermore confirmed by p24 ELISA in blood BDCA-1^+^ DCs (S1B Fig).

Next, we tested infection of DCs using differentially opsonized GFP-tagged HIV-1 preparations carrying Vpx or not. Confocal as well as FACS analyses revealed, that the Vpx-carrying positive controls (HIV*Vpx, HIV*Vpx-C) as well as HIV-C showed higher percentages of GFP^+^DCs compared to HIV or iDC controls (Fig 1B and S1C Fig). In case of confocal microscopic analyses, 40 cells per condition were used to calculate percentages of GFP^+^ cells. We detected a higher up-take of HIV*Vpx (48% GFP^+^ cells), HIV-C (69% GFP^+^ cells) and HIV*Vpx-C (80% GFP^+^ cells) compared to HIV (19% GFP^+^ cells). Also FACS analyses revealed about twice as much DCs infected using HIV-C, HIV*Vpx or HIV*Vpx-C compared to non-opsonized HIV (S1C Fig). The lower number of infected cells compared to confocal microscopic analyses is due to the lower MOI used for FACS analyses (MOI 1 for confocal analyses vs. 0.05 for FACS analyses), which was chosen to monitor infection kinetics also by flow cytometry. In summary, here we found by various methods, that replication mediated by C-opsonized HIV-1 is significantly higher in monocyte-derived DCs and also in blood DCs compared to low-level DC infection using non-opsonized HIV.

### Efficient DC infection by HIV-C is associated with significantly higher phosphorylation of SAMHD1

**Fig 2:**
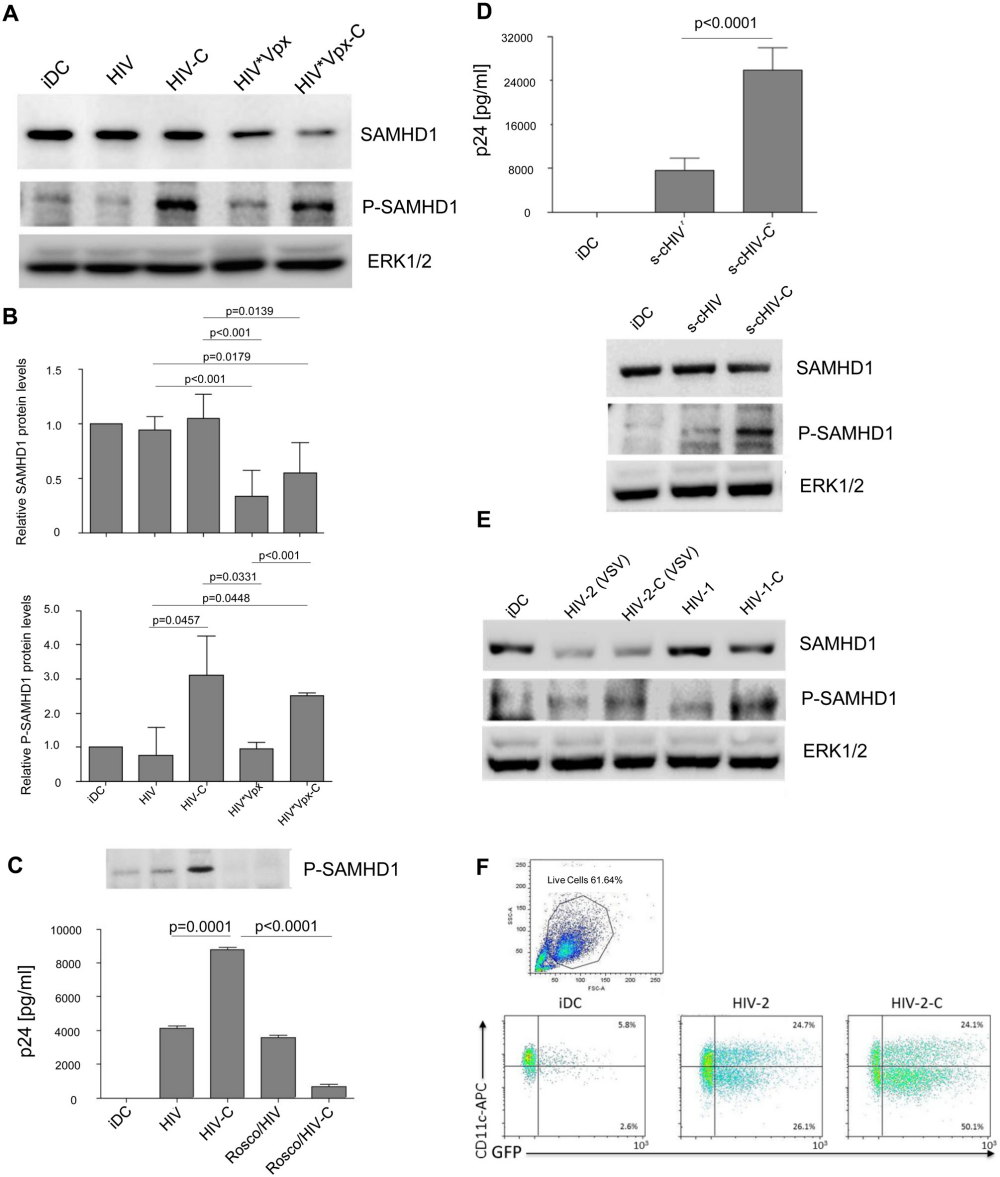
SAMHD1 is highly phosphorylated in HIV-C-DCs. *(A) 1* ^*st*^ *panel*: SAMHD1 protein expression is unaltered in DCs incubated with HIV or HIV-C compared to iDCs (lanes 1–3), while degradation of SAMHD1 can be detected after 24h when cells were incubated with HIV*Vpx and HIV*Vpx-C (lanes 4 and 5). *2* ^*nd*^ *panel*: Compared to iDCs (lane 1), and DCs exposed to non-opsonized HIV (HIV, lane 2) or HIV*Vpx (HIV*Vpx, lane 4), SAMHD1 phosphorylation at SAMHD1 residue T592 is highly increased after 4h in C-opsonized HIV and HIV*Vpx-DCs (HIV-C and HIV*Vpx-C, lanes 3 and 5). *3* ^*rd*^ *panel*: Protein expression of non-phosphorylated ERK1/2 was used as control for proper protein loading of the samples. Western Blots were performed using lysates of DCs loaded with differentially opsonized HIV (HIV and HIV*Vpx) for 24h from 5 donors and one representative example is shown. *(B)* SAMHD1 (left chart) and P-SAMHD1 (right chart) protein expression values (from Western blot experiments) were normalized from 3 donors as stated in the Results section by ImageJ quantification. Protein expression of ERK1/2 was used as loading control and additionally relative comparison to iDC protein expression was performed. *(C) Inhibition of SAMHD1 phosphorylation abrogates HIV-C-mediated DC infection*. DCs were incubated or not for 24h with the specific CDK2 inhibitor Roscovitine (50μM) before infection with HIV or HIV-C for another 24h. Immuno-blot analyses of iDCs, HIV-DCs, HIV-C-DCs, Roscovitine-HIV-DCs and Roscovitine-HIV-C-DCs were performed (upper panel). No SAMHD1 T592 phosphorylation signal was detected in DCs pre-treated with Roscovitine (lane 4, 5), while again C-opsonized HIV (HIV-C) mediated SAMHD1 T592 phosphorylation (lane 3). Additionally, infection assays were performed in absence and presence of Roscovidine. As shown by p24 ELISA on the chart, HIV-C significantly enhanced DC infection compared to non-opsonized HIV, while pre-incubation with Roscovitine significantly blocked HIV-C-mediated DC infection. For these analyses, cells from two donors and three different virus strains were used. *(D) Productive DC infection and SAMHD1 phosphorylation are also enhanced using complement-opsonized single-cycle HIV-1*. Analyses of infection and SAMHD1 phosphorylation were repeated in three independent experiments (in triplicates for infection assays) using single-cycle HIV-1, which was opsonized or not with complement (s-cHIV, s-cHIV-C). These analyses revealed that the highly significant (p<0.0001) enhancement of DC infection by HIV-C does not depend on multiple rounds of infection, but is also achieved using s-cHIV-C (left). As observed with replicating HIV, also s-cHIV only caused a low-level productive infection in DCs (left). Furthermore, s-cHIV-C mediated SAMHD1 phosphorylation in contrast to the non-opsonized HIV-1 preparation (right). *(E) HIV-2 (VSV) degrades SAMHD1*. *1* ^*st*^ *panel*: SAMHD1 protein expression is similar in control DCs (iDC; lane 1), or non- and C-opsonized HIV-1 (HIV-1, HIV-1-C; lanes 4 and 5) after 24h. In contrast, non-opsonized and C-opsonized HIV-2 (HIV-2, HIV-2-C; lanes 2 and 3) caused degradation of total SAMHD1 protein expression levels. *2* ^*nd*^ *panel*: T592 phosphorylation of SAMHD1 was detected after 3h in C-opsonized HIV-1 loaded DCs (HIV-1-C; lane 5) and at a lower level in C-opsonized HIV-2 treated DCs (HIV-2-C; lane 3). Similar to previous results (Fig 2A) no phosphorylation of SAMHD1 was observed in immature DCs (iDC; lane 1) as well as in cell lysates from non-opsonized HIV-1 or HIV-2 treated DCs (HIV-1, HIV-2; lanes 2 and 4). Results of one representative donor are shown in the figure. The experiment was repeated in 3 different donors. *(F) HIV-2 (VSV) and HIV-2-C (VSV) efficiently infect DCs*. FACS analyses of live, HIV-2-exposed DCs revealed productive infection with both HIV-2 (VSV) and HIV-2-C (VSV). Nevertheless, C-opsonization of HIV-2 mediated a 1.5-fold enhancement of infection in three independent donors. One representative FACS analysis is depicted.

Recently, SAMHD1 was identified in macrophages, DCs and resting CD4^+^ T cells and shown to block infection with HIV-1 [17, 19, 25, 26]. Although expressed in non-cycling and cycling cells, the antiviral activity of SAMHD1 is only observed in non-cycling cells and depends on phosphorylation status of position T592, which negatively regulates the RNase activity of SAMHD1 [20, 23]. T592 phosphorylation was shown to abolish the restrictive capacity of SAMHD1 and to make cells permissive to HIV-1 infection [20, 21, 23]. Therefore, we next explored, whether SAMHD1 is degraded or phosphorylated upon exposure of DCs to HIV-C. We treated DCs with HIV, HIV-C, and HIV*Vpx or HIV*Vpx-C as positive controls for SAMHD1 degradation, and studied SAMHD1 protein expression and phosphorylation status by immunoblotting. While similar amounts of SAMHD1 protein were expressed in iDCs and HIV- or HIV-C-exposed DCs (Fig 2A, 1^st^ panel), SAMHD1 was degraded by HIV coupled to simian Vpx (18, 19) and independent on the opsonization pattern (Fig 2A, 1^st^ panel). Since the Vpx-containing particles were assembled from two constructs as described in the Methods section, residual SAMHD1 expression in Vpx-treated cells is probably due to HIV particles lacking the Vpx construct.

In contrast to iDC controls or DCs exposed to HIV or HIV*Vpx (Fig 2A, 2^nd^ panel), HIV-C and HIV*Vpx-C mediated a significantly increased SAMHD1 phosphorylation in DCs at T592 (Fig 2A, 2^nd^ panel). Fig 2B summarizes the relative SAMHD1 and P-SAMHD1 protein levels from 5 donors. Using HIV-C exposed DCs we here illustrated a significantly higher SAMHD1 phosphorylation at T592, but no degradation of SAMHD1.

### SAMHD1 phosphorylation is a prerequisite for efficient DC infection using HIV-C

To further confirm our findings, that SAMHD1 phosphorylation is responsible for the efficient productive infection of DCs with HIV-C, we next pre-incubated DCs with the potent CDK2 inhibitor Roscovitine before exposure to virus. Among others, CDK2 was recently described to be a regulator of SAMHD1 phosphorylation [27, 28]. Pre-incubation of DCs with Roscovitine prior to HIV loading resulted in a complete inhibition of SAMHD1 phosphorylation (Fig 2C, immunoblot) and a significant impairment in productive infection with HIV-C, while the low-level HIV-infection was not affected (Fig 2C, chart). Inhibition of SAMHD1 phosphorylation by Roscovitine was repeated using cells from 3 independent donors and results are summarized in Fig 2C (chart). We here clearly illustrate that phosphorylation of SAMHD1 T592 by HIV-C but not SAMHD1 degradation is a prerequisite for efficient DC infection.

### HIV-C-mediated enhancement of infection and SAMHD1 phosphorylation also occur using an opsonized single-cycle virus preparation

To explore, if the 5-fold increase of productive DC infection with HIV-C results from multiple rounds of DC infection or from a single round only, we performed DC infection experiments using a single-cycle (s-c) virus preparation, which was opsonized or not with C. These analyses revealed that also the C-opsonized s-cHIV-1 preparation significantly enhanced DC infection compared to its non-opsonized counterpart, since a 4-fold increase in productive infection was measured using s-cHIV-C (Fig 2D, chart). Additionally, also s-cHIV-C induced a significantly higher phosphorylation of SAMHD1 T592, while in DCs exposed to s-cHIV this residue was not phosphorylated (Fig 2D, immunoblot). Therefore, we here showed that complement-opsonization of HIV-1 significantly enhances DC infection and SAMHD1 phosphorylation independent on the use of single-cycle or replicating virus.

### SAMHD1 is phosphorylated to lower levels also in DCs exposed to HIV-2-C

In additional experiments, we compared effects of complement opsonization on SAMHD1 degradation and phosphorylation in DCs between HIV-1 and HIV-2 (expressing Vpx) as well as DC infection using differentially opsonized HIV-2 (VSV). We used a VSV-pseudotyped HIV-2 preparation, since as already described in the literature [29, 30] we did not obtain a significant infection using the HIV-2 WT preparation. HIV-2 (VSV) degraded SAMHD1 protein in DCs independent on opsonization contrary to HIV-1 (Fig 2E, upper panel). Comparable to complement-opsonized Vpx-carrying HIV-1 preparations, also HIV-2-C phosphorylated SAMHD1 at the T592 residue (Fig 2E, middle panel), thus increasing productive HIV-2 DC infection about 1.5-fold as measured by flow cytometry (Fig 2F; 42.4% HIV-2- vs. 65.8% HIV-2-C-infected live DCs; background subtracted). Therefore, DC infection with HIV-2 is enhanced by complement opsonization, too, albeit to lower levels.

### HIV-C-mediated SAMHD1 phosphorylation of DCs is not due to increased viral entry, and enhanced DC infection is due to more efficient transcription of viral RNA

To illustrate, if the complement-mediated effect is due to higher viral entry, we determined HIV and HIV-C fusion with DCs using the Vpr-blam assay and various concentrations of virus (0-5-50-250-500 ng p24/ml). These experiments were repeated using cells from 3 donors. They revealed that similar amounts of HIV and HIV-C enter DCs independent on the opsonization pattern and on the virus concentration used (S2A Fig), thereby not explaining the enhanced SAMHD1 phosphorylation and HIV-1-replication due to increased viral entry of HIV-C. To further characterize the enhanced capacity of HIV-C to infect DCs, we monitored full-length and integrated HIV-1 DNA in HIV- and HIV-C-loaded DCs. These assays revealed that 48h post infection both, relative expression levels of full-length (FL) HIV-DNA in the cytoplasm (p = 0.0044) as well as integrated HIV-DNA in the nucleus (>100-fold) are much higher in HIV-C- compared to HIV-DCs (S2B Fig). Increased viral entry and internalization by HIV-C is not the pre-requisite for the observed effects respecting SAMHD1 phosphorylation and replication. Enhancement of viral replication in HIV-C-DCs already occurs in the cytoplasm as analyzed by significantly increased expression of HIV full-length DNA and enhanced integration of the viral DNA into the host genome, thereby confirming the inhibitory activity of C-opsonized HIV-1 on the restrictive capacity of SAMHD1.

### HIV-C-exposed DCs exhibit a higher maturation and expression of co-stimulatory markers

**Fig 3:**
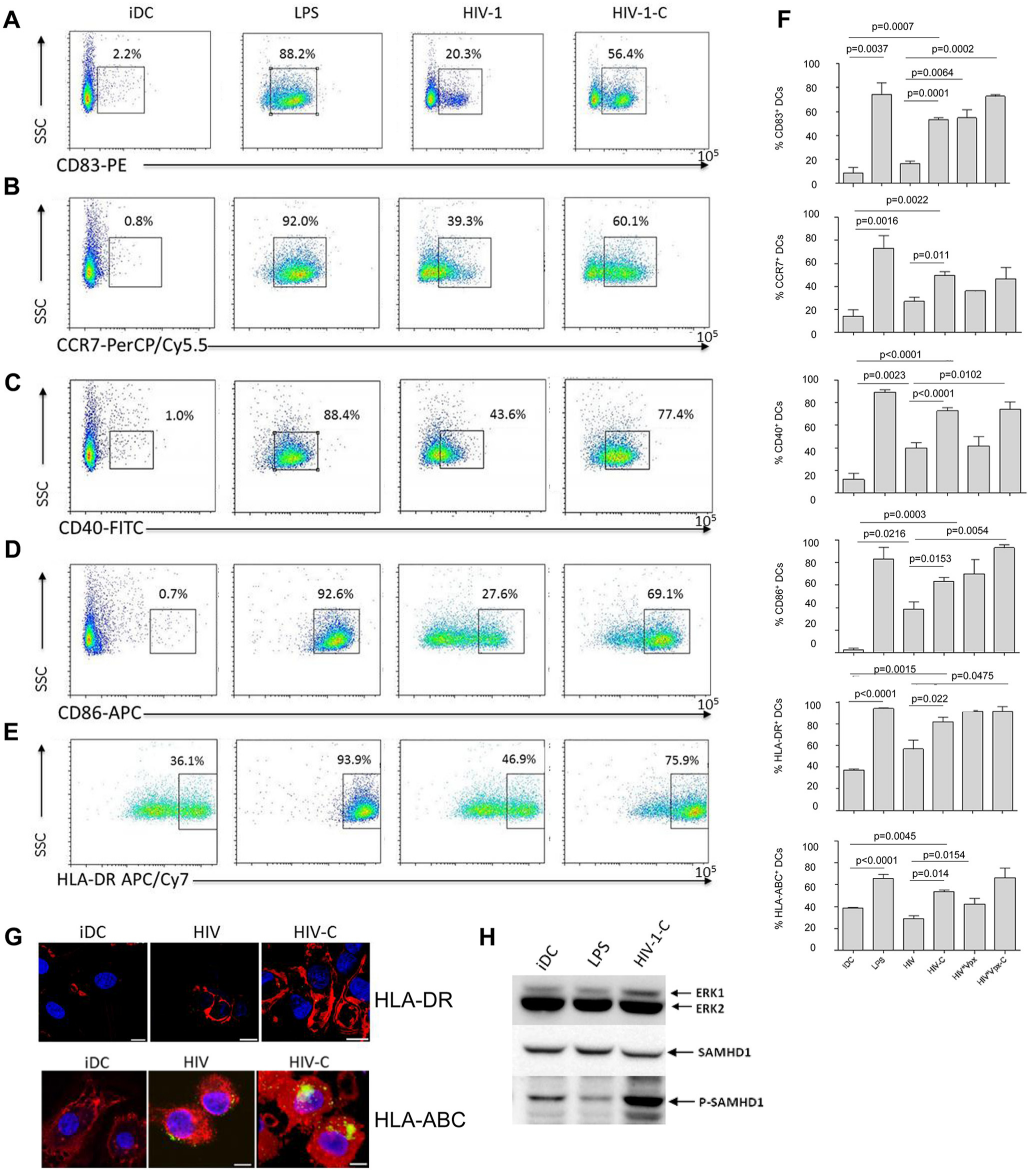
Complement-opsonization of HIV stimulates DC maturation and activation. DCs exposed for 48h to LPS (LPS), non-opsonized HIV (HIV-1) and C-opsonized HIV-1 (HIV-1-C) showed a highly activated and mature phenotype compared to control DCs (iDC) as measured by FACS analyses using *CD83 (A)*, *CCR7 (B)*, *CD40 (C)*, *CD86 (D) and HLA-DR (E)*. Immature DCs (iDC) and LPS-matured DCs (LPS) served as negative and positive controls for DC maturation, respectively. FACS analyses were repeated 5 times using DCs from different donors and Fig 3A–3E show representative dot plots from one donor. *(F) Summary of flow cytometric analyses of maturation*, *antigen-presentation and co-stimulatory markers from 5 donors*. Percentages of cells positive for expression of CD83 (1^st^ chart), CCR7 (2^nd^ chart), CD40 (3^rd^ chart), CD86 (4^th^ chart), HLA-DR (5^th^ chart) and HLA-ABC (6^th^ chart) are depicted. Significantly higher percentages of mature, activated DCs were measured in DCs loaded with HIV-C compared to HIV. Similar results were analyzed respecting Vpx-carrying HIV preparations. Also, C-opsonized HIV*Vpx increased DC maturation and activation to higher levels than non-opsonized HIV*Vpx. *(G) Higher expression of HLA-DR and HLA-ABC in HIV-C DCs*. In addition to flow cytometric analyses expression of HLA-DR was measured by confocal microscopy. HLA-DR (red) was higher in C-opsonized HIV loaded DCs (HIV-C, right) compared to non-opsonized HIV (HIV, middle) or iDC controls (left). Cell nuclei were stained with Draq5 (blue) and 40 cells were counted per condition. Furthermore, confocal microscopic analyses of HLA-ABC underlined the C-mediated DC activation compared to HIV-DCs or iDCs. Virus: green, HLA-ABC: red, nucleus: blue; Scale: 10 μm. *(H) LPS does not mediate SAMHD1 phosphorylation in DCs*. To exclude that the SAMHD1 phosphorylation is due to maturation of DCs, DCs exposed to LPS were analyzed for phosphorylation at the T592 residue of SAMHD1. As positive controls for SAMHD1 phosphorylation, DCs were also loaded with HIV-C. Compared to iDC control cells, LPS-stimulated DCs showed a weaker phosphorylation signal of SAMHD1 (middle panel) while HIV-C caused a high phosphorylation at this residue. The experiment was repeated using differentially stimulated DCs from 5 different donors.

To identify, if the enhanced DC infection status by HIV-C treatment is associated with a higher maturation, we next studied characteristic DC maturation markers and co-stimulatory molecules by multicolor FACS analyses. We found that CD83 (Fig 3A and 3F, first chart), CCR7 (Fig 3B and 3F, second chart), CD40 (Fig 3C and 3F, third chart), CD86 (Fig 3D and 3F, fourth chart), HLA-DR (Fig 3E and 3F, fifth chart) and HLA-ABC (Fig 3F, sixth chart) were expressed to significantly higher percentages on DCs exposed to HIV-C compared to HIV-DCs (Fig 3A–3F). LPS-stimulated DCs and iDCs served as positive and negative controls, respectively (Fig 3A–3F). Also differentially opsonized Vpx-carrying HIV particles caused a significantly enhanced expression of CD83, CD86 and HLA-DR (Fig 3F). With respect to CCR7, CD40 and HLA-ABC up-regulation, HIV*Vpx preparations expressed a similar pattern as HIV-DCs (Fig 3F). The percentages of up-regulation of co-stimulatory and maturation markers varied among donors; nevertheless the C-enhancing effects were observable in all donors tested and a summary of 5 donors showing maturation/co-stimulatory/antigen-presenting DC markers after addition of LPS, HIV, HIV-C, HIV*Vpx or HIV*Vpx-C compared to iDCs is depicted in Fig 3F. In addition, we detected a higher up-regulation of HLA-DR as well as HLA-ABC upon exposure of DCs to HIV-C compared to HIV or iDC controls by confocal microscopy (Fig 3G). The results obtained herein clearly indicate that DCs illustrate a significantly higher maturation and expression of co-stimulatory molecules when exposed to HIV-C compared to non-opsonized HIV particles. To rule out that complement itself is responsible for DC maturation, we furthermore incubated DCs using non-opsonized and C-opsonized latex beads and analyzed these cells for up-regulation of CD83 (S3 Fig). We found that Beads and Beads-C slightly activated DCs (6.2% and 4.1% CD83^high^ cells) compared to iDC controls (2.5% CD83^high^ cells), but to similar amounts. Therefore, we could exclude that the DC maturation by HIV-C is only due to the adjuvant effect of complement.

### SAMHD1 phosphorylation is not due to DC maturation

To rule out that SAMHD1 phosphorylation by HIV-C occurs non-specifically upon DC maturation, we next incubated DCs with either LPS (100ng/ml), HIV-C or left them untreated (iDCs). These experiments revealed that upon stimulation with LPS, SAMHD1 phosphorylation in DCs was decreased (Fig 3H) or similar compared to iDCs. In contrast, complement-opsonization of HIV-1 initiated a highly enhanced phosphorylation of this protein in DCs (Fig 3H). As observed before, total SAMHD1 protein levels were similar under all conditions. Therefore, SAMHD1 is phosphorylated in DCs due to complement-opsonization of HIV-1 and not due to DC maturation.

### HIV-C-exposed DCs display a higher antiviral potential

**Fig 4:**
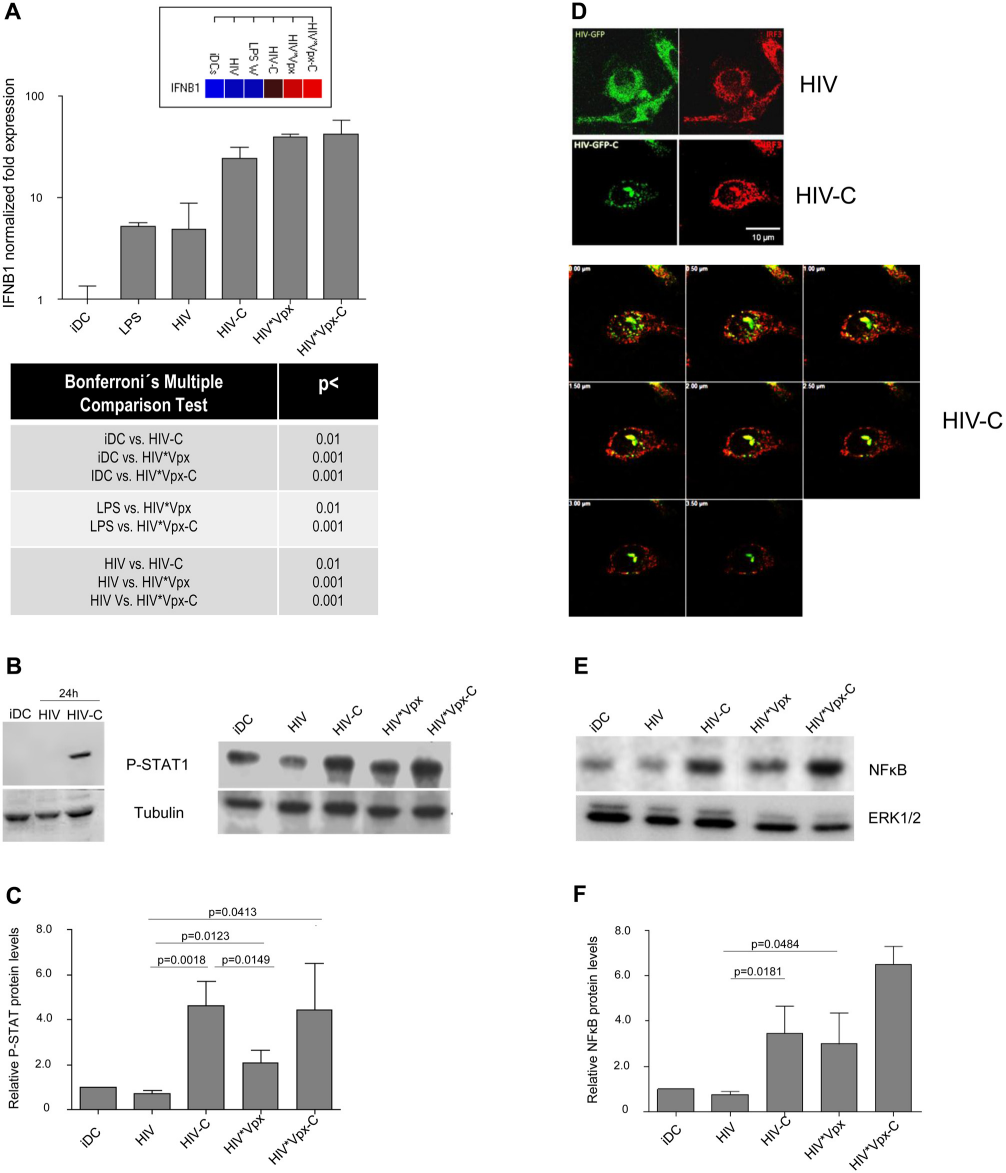
HIV-C activates type I IFN responses in DCs. *(A)* mRNA expression of IFNB1 is induced to significantly higher levels in DCs loaded with C-opsonized or Vpx-containing HIV (HIV-C; HIV*Vpx- or HIV*Vpx-C) in contrast to iDCs or non-opsonized HIV (HIV). mRNA expression was relatively quantified using GAPDH gene expression as reference and the ΔΔC_t_ method. Relative expression levels displayed as a cluster gram are depicted above the bar chart with blue/black representing lowest expression and red highest expression. Tabular statistics results from the same donor are illustrated below chart (One-way ANOVA using Bonferroni post-test for multiple comparisons). Relative quantification by RT-PCR was performed using cells from 4 donors (triplicates) and one representative donor is shown in the figure. *(B)* Phosphorylation of STAT1 (pSTAT1) was induced in DCs exposed to C-opsonized HIV after 24h (HIV-C; left, lane 3), while no STAT1 activation was detected in unstimulated DCs (iDC; left, lane 1) or DCs exposed to non-opsonized HIV (HIV; left, lane 2). Not only C-opsonized HIV (HIV-C; right, lane 3), but also C-opsonized Vpx-carrying HIV (HIV*Vpx-C; right, lane 5) activated STAT1 phosphorylation to higher levels compared to their non-opsonized counterparts (right, lanes 2 and 4) or iDCs (right, lane 1). Tubulin expression was used as control for proper loading and cell lysates from 4 different donors were analyzed. *(C)* pSTAT1 protein expression from 4 donors were summarized using ImageJ quantification. C-opsonization of HIV preparations (HIV-C, HIV*Vpx-C) led to significantly higher STAT1 activation in DCs after 24h compared to non-opsonized HIV or Vpx-carrying HIV (HIV, HIV*Vpx). *(D)* IRF3 (red) was translocated to the nucleus in DCs exposed to C-opsonized HIV (HIV-C; left, lower panel), whereas this translocation was not detected by confocal microscopy in DCs loaded with non-opsonized HIV (HIV, left upper panel). IRF3 (red) translocation is depicted by z-stack (0.5 μm steps, right) of DCs loaded with C-opsonized HIV (HIV-C; green, right). *(E)* Activation of NFkB was detected by Western blot analyses using an antibody directed against the nuclear localization sequence of human p65, therefore selectively binding to the activated form of NFκB. NFκB is strongly activated in DCs loaded with HIV-C and HIV*Vpx-C (lanes 3 and 5). A slight activation of NFκB is observed in HIV*Vpx-treated DCs (lane 4) compared to iDCs (lane 1) or HIV-DCs (lane 2). Western Blot analyses were repeated three times. *(F)* NFκB levels were relatively quantified as described in Fig 2B using values from three different donors and one-way ANOVA using Bonferroni post-test for multiple comparisons.

After proving that HIV-C mediates SAMHD1 phosphorylation in DCs, which was associated with a higher susceptibility to HIV-1 and higher maturation, we wanted to see, if DCs also signal the presence of this virus preparation more efficient. Firstly, we studied humoral antiviral immune responses and found that significantly higher IFNB1 mRNA levels were induced by HIV-C, HIV*Vpx and HIV*Vpx-C (Fig 4A and S4A Fig). iDCs and LPS-treated DCs were used as controls (Fig 4A and S4A Fig). Since type I IFNs mediate activation of STAT1, we next studied STAT1 phosphorylation in DCs treated with HIV or HIV-C. By immunoblotting we found that STAT1 was only activated in DCs exposed to HIV-C for 24h (Fig 4B, left), while not in iDCs or HIV-DCs (Fig 4B, left). Furthermore, iDCs were also exposed to HIV*Vpx and HIV*Vpx-C, which also induced higher STAT1 phosphorylation levels (Fig 4B, right). Despite donor-specific variations in basal STAT1 phosphorylation levels in iDCs (Fig 4B left vs. right), we illustrated that HIV-C mediated significantly higher STAT1 activation in DCs from 5 different donors as summarized in Fig 4C. Enhanced expression levels of genes associated with type I IFN signaling and T cell stimulatory capacity were furthermore detected by microarray analyses (S1 Text and S4B Fig). In particular, ISGs associated with T cell activating capacity (CXCL9, CXCL10, CXCL11), were up-regulated in HIV-C compared to HIV-DCs (S4B Fig). Type I IFN-associated genes were recently described in macrophages to be initiated by HIV-1 capsid mutants in contrast to the WT virus, which replicates in these cells without stimulating innate immunity [31]. In contrast to DCs exposed to non-opsonized HIV, IRF3 (Fig 4D) and NFκB (Fig 4E and 4F) were translocated to the nucleus in DCs loaded with HIV-C. IRF3 translocation to the nucleus was observed in 63% of HIV-C DCs compared to 13.5% translocation in HIV-DCs. A representative example for HIV or HIV-C is depicted in Fig 4D. Activation of NFκB in HIV-C-, HIV*Vpx- and HIV*Vpx-C-, but not HIV-DCs was detected by immunoblotting using an NFkBP65-Ab directed against the nuclear localization sequence (NLS) of human p65, therefore selectively binding to the activated form of NFκB (Fig 4E and 4F). These analyses altogether emphasize the important role of complement to efficiently signal the viral presence to DCs, thereby initiating an efficient antiviral immune potential.

### HIV-C-exposed DCs reveal a higher T cell stimulatory capacity and antiviral potential

**Fig 5:**
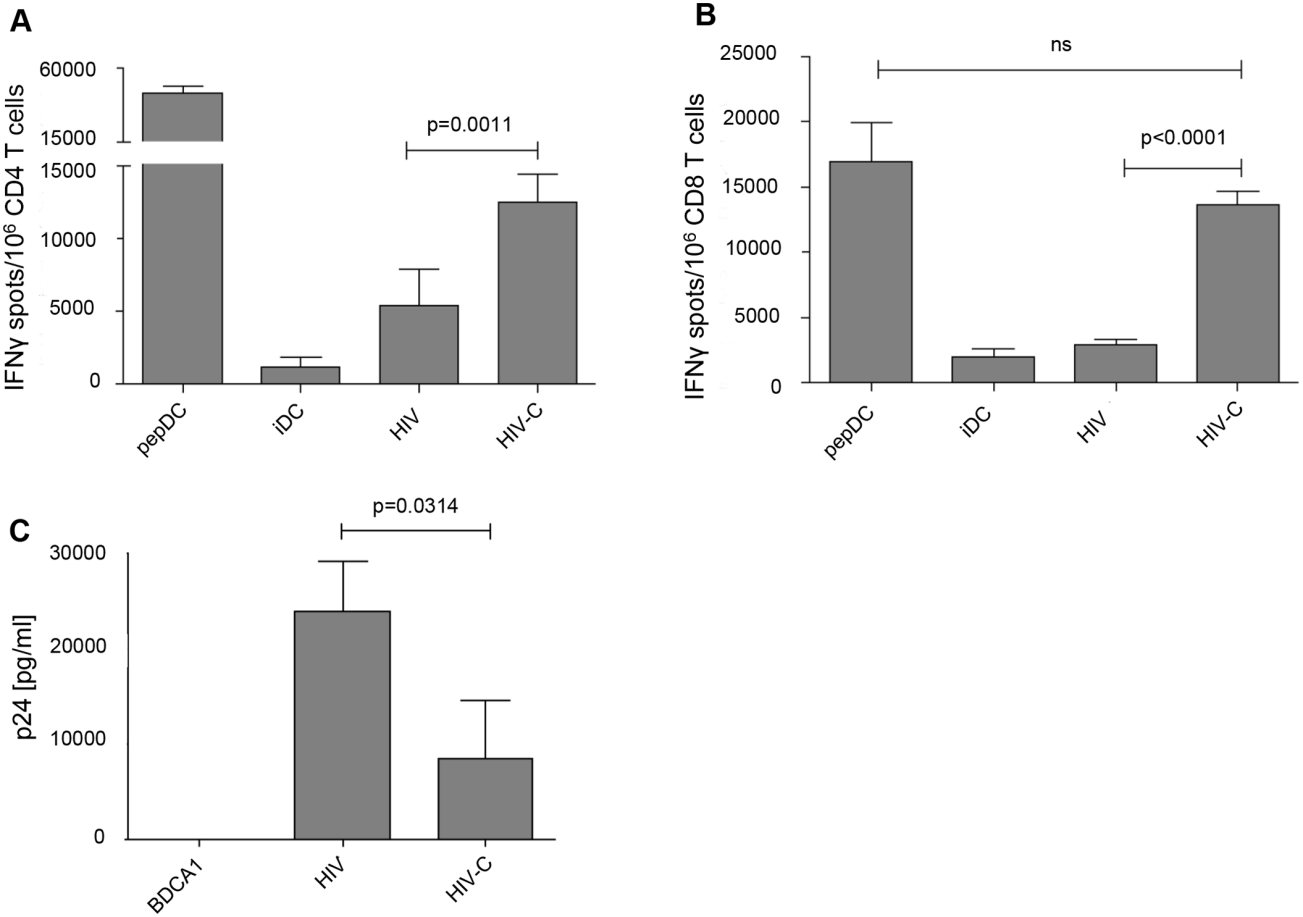
HIV-C-DCs show a significantly higher activation of specific CD4^+^ and CD8^+^ T cell clones and higher suppression of viral replication. IFNγ induction in both CD4^+^ *(A)* and CD8^+^ *(B)* T cell clones by C-opsonized HIV exposed DCs (HIV-C) was significantly stronger than that of non-opsonized HIV loaded DCs (HIV-DCs; p = 0.0011 for CD4^+^ T cell clones, p<0.0001 for CD8^+^ T cell clones). Untreated DCs (iDC) served as negative control and as positive controls specific peptide-loaded DCs for CD4^+^ and CD8^+^ T cell clones were used (pepDC). IFNγ Elispots of differentially stimulated CD4^+^ and CD8^+^ T cell clones were repeated using HLA-matched DCs from three donors exposed to differentially opsonized HIV-1 preparations (HIV, HIV-C). *(C)* Co-cultures of HIV- or HIV-C-exposed BDCA1^+^ DCs and autologous T cells revealed that productive infection of HIV-1 was significantly suppressed, when complement-opsonized HIV was used for loading of DCs compared to HIV-BDCA1^+^ DCs (p = 0.0314). A summary of p24 ELISA values on day 7 pI from supernatants of 3 different donors performed in triplicates is depicted. p24 values from HIV- and HIV-C- loaded BDCA1^+^ DC/CD4^+^ T cell co-cultures were analyzed using an unpaired Student´s t test.

Next, we studied the antigen-presenting capacity of differentially loaded DCs using HLA-matched HIV-specific T cell clones. HLA-matched DCs exposed for 24h to HIV or HIV-C, were co-cultured for another 24h with HIV-specific CD4^+^ or CD8^+^ T cell clones. IFN Elispots (Fig 5A and 5B) were used to measure activation of specific clones. We found that significantly higher IFN-γ spots were induced when using HIV-C-DCs for stimulation of both CD4^+^ (p = 0.0011, Fig 5A) and CD8^+^ T cell clones (p<0.0001, Fig 5B). Untreated (iDC) and peptide-exposed DCs (pepDC) were used as negative and positive controls, respectively. To investigate the antiviral potential of differentially loaded DCs, we also co-cultured HIV- and HIV-C-exposed BDCA1^+^ DCs with autologous T cells and monitored productive infection using p24 ELISA. These analyses revealed that HIV-C-BDCA1^+^ DCs significantly suppressed virus production in these co-cultures compared to HIV-infected BDCA1^+^ DCs (Fig 5C). Non-infected DCs were used as negative controls (Fig 5C). Thereby, HIV-C-exposed DCs exert a significantly higher antigen-presenting capacity for both, CD4^+^ and CD8^+^ T cells, and suppress virus production in infected co-cultures. These data indicate that complement opsonization together with DCs play a central role in virus control especially during the acute phase of infection.

## Discussion

**Fig 6:**
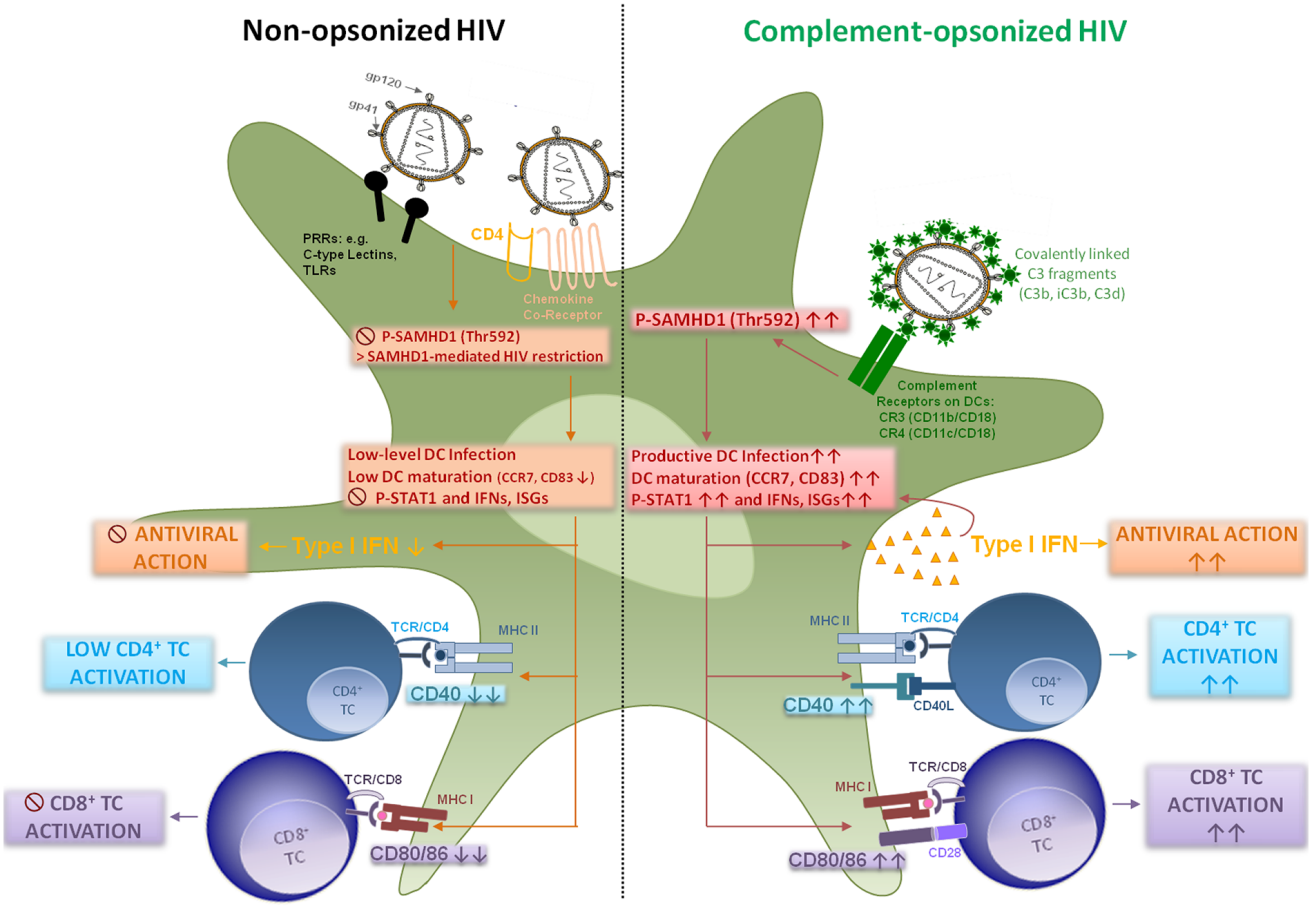
Graphical summary—HIV-C modulates dendritic cell function. In this study, we found that SAMHD1 restriction in DCs is overcome, when HIV-1 is opsonized by covalently linked C fragments. Ligation of C3-coated HIV-1 to CR3 and CR4 mediates phosphorylation of SAMHD1 at residue Thr592. Phosphorylation at this particular SAMHD1 residue allows HIV-1 infection as shown in proliferating, permissive cells. By-passing HIV-1 restriction caused a significantly enhanced productive infection of myeloid and blood DCs. The increased DC infection was associated with enhanced maturation as shown by CD83 and CCR7 up-regulation, enhanced co-stimulatory function as measured by CD40 and CD86 expression as well as up-regulation of type I IFNs and ISGs. Due to these changes, DCs, which were exposed to complement-opsonized HIV-1, mediated significantly higher HIV-specific CD4^+^, CD8^+^ T cell and antiviral immune responses compared to HIV-loaded DCs.

We here give insight into a substantial novel way of dendritic cell modulation at least during acute HIV-1 infection by triggering integrin receptor signaling. We found, that complement-opsonization of the virus is able to relieve SAMHD1 restriction in DCs due to T592 phosphorylation, to initiate strong maturation and co-stimulatory capacity of DCs and to stimulate efficient cellular and humoral antiviral immune responses (Fig 6, Graphical Summary). Since we recently illustrated an important role for complement in facilitating antigen presentation by DCs to stimulate the activation, expansion, and differentiation of retrovirus-specific CD8^+^ T cells *in vitro* and *in vivo* [24, 32], we were now aiming to elucidate the mechanisms of this better capacity of HIV-C-exposed DCs to activate T cells. HIV-1 evolved mechanisms to avoid complement-mediated destruction by incorporating regulators of complement-activation during the budding process and additionally binding the fluid phase factor H [33, 34]. Thus, only low levels of virus particles are lysed by action of complement [33]. Additionally, the virus displays a C1q-binding domain in its envelope protein, thereby spontaneously activating the classical complement pathway upon entry into the host—even in the absence of HIV-specific antibodies [8, 35]. Because of spontaneous activation of C and protection against viral lysis, C3-coated HIV particles accumulate already in semen or immediately after entry of viruses via mucosal surfaces [36, 37]. Since C3b and its fragments are covalently linked to the viral surface and surround the virus like a halo, *in vivo* C3-coated particles will more likely interact with CR3 and CR4 abundantly expressed on DCs [9, 38].

DCs are relatively resistant to productive infection with HIV-1 due to several restriction mechanisms [20, 21, 39–41]. Manel et al. showed that low DC infection with HIV-1 was bypassed by co-delivery of HIV-2 or SIV Vpx, which significantly enhanced DC infection, activation and induction of an efficient type I IFN response [4]. About one year after this finding, SAMHD1 was characterized in non-cycling (non-permissive) cells, i.e. DCs, primary macrophages or quiescent CD4^+^ T cells as the important HIV-1 restriction factor reducing cellular dNTP levels and counteracted by Vpx [19–21, 41]. Several groups pointed out the critical role of T592 phosphorylation respecting the retroviral restriction ability of SAMHD1 in permissive cells [20–23, 42]. Very recently, Ryoo et al. revealed that the RNase activity of SAMHD1 was required for HIV-1 restriction in myeloid cells, which was shown to be negatively regulated by T592 phosphorylation in permissive cells [23]. We and others earlier found a significant enhancement of productive DC infection using HIV-C [7, 9, 43–45], which we also demonstrated herein using various methods as well as complement-coated Vpx-containing HIV-1. This enhanced infection of DCs using HIV-C was accompanied by a highly phosphorylated status of SAMHD1 T592, which was associated with relieved HIV-1 restriction and higher induction of cellular and type I IFN immune responses. Phosphorylation of SAMHD1 was not induced because of higher viral entry by HIV-C as shown using the Vpr-blam assay, and SAMHD1 was also phosphorylated at the T592 residue using C-opsonized single-round HIV-1. Blocking SAMHD1 phosphorylation resulted in a significantly impaired HIV-1 replication in DCs. CR3 (CD11b/CD18) and CR4 (CD11c/CD18) are integrin receptors, which are composed of the common leukocyte-specific β2 integrin CD18 and the specific αM and αX integrin chains (CD11b or Mac-1 and CD11c). Incorporation of HIV-C via CRs expressed on DCs might protect the virus from immediate and extensive degradation of internalized particles in intracellular compartments, as shown for non-opsonized HIV-1 [46–48]. Beside the significantly increased phosphorylation of SAMHD1 in DCs, HIV-C also induced a powerful activation of innate DC responses. This was characterized by significant up-regulation of maturation (CD83, CCR7), antigen-presenting (HLA-DR, HLA-ABC) and co-stimulatory (CD40, CD86) markers and boost in type I IFN responses. To exclude that SAMHD1 T592 phosphorylation occurs only due to DC maturation and not specifically due to HIV-C, we analyzed SAMHD1 phosphorylation also in DCs exposed to LPS—these analyses revealed that merely HIV-C, but not LPS, caused SAMHD1 phosphorylation in DCs. Manel et al. reported that delivery of SIV-VLPs abrogated HIV-1 restriction mechanisms in human DCs, up-regulated co-stimulatory and type I IFN functions, thus providing an ameliorated antigen presentation [4]. We here demonstrate that complement-opsonized HIV also activated STAT1, NFκB and IRF3. Additionally, type I IFNB1 mRNA significantly increased in HIV-C-, HIV*Vpx- and HIV*Vpx-C-DCs, which all caused a considerably higher HIV-1-replication in DCs. This type I IFN induction was not observed in iDCs, LPS- or HIV-DCs. Furthermore, by microarray analyses, we also found increased expression of type I IFN genes and in particular genes associated with T cell stimulation in HIV-C-exposed DCs in contrast to HIV-DCs. Our findings are in substantial disagreement to a manuscript recently published by Ellegård et al. [49], who showed that complement opsonization of HIV-1 resulted in a decreased antiviral immune response in DCs. Similar to our previous works [7, 9, 24, 32], the authors illustrated herein and in an earlier study that DCs are infected to significantly higher levels if the virus was complement-opsonized [45, 49] and they furthermore described an enhanced IRF3 activation in DCs upon HIV-C treatment [49]. We also observed enhanced productive infection of HIV-C-exposed DCs and an increased IRF3 activation, but in contrast to Ellegård et al. [49] we found an enhanced type I IFN response. Type I IFN responses after viral infection were described to require the activation and translocation of IRF3 [50], which we and Ellegård et al. [49] illustrated. Manel et al. [4] nicely showed that, if restriction to infection was relieved by addition of Vpx, newly synthesized HIV-1 capsids induced efficient DC activation and antiviral immune responses via IRF3 and CYPA. Also Su et al. [51, 52] very recently demonstrated that if SAMHD1 levels in DCs were decreased by co-culture with CD4^+^ T cells, an enhanced HIV-1 replication in DCs was observed accompanied by DC maturation and increased type I IFN secretion from the cells. These studies are in accordance to our study where we, too, found that enhancement of DC infection—in our case using HIV-C—mediated a significant up-regulation of maturation, antigen-presenting and co-stimulatory markers on DCs accompanied by efficient induction of type I IFN responses.

Of note, we earlier found a significantly higher capacity of HIV-C-exposed DCs to prime CD8^+^ T cell expansion and activation of HIV-specific T cell clones compared to DCs loaded with non-opsonized HIV-1 [24, 32]. The higher activation of HIV-specific CD8^+^ T cell clones was also observed herein. Furthermore we could show an increased stimulation of HIV-specific CD4^+^ T cell clones by DCs exposed to HIV-C, which is in contradiction with Tjomsland et al. [53]. This discrepancy can be explained by the difference that we and others [54, 55] perform all experiments using CD4^+^ T cell clones with chemically inactivated virus or in the presence of HIV blockers to prevent viral replication. We found that not only infectious HIV-C, but AT2-inactivated HIV-C up-regulated DC maturation and co-stimulatory markers to significantly higher levels compared to AT2-HIV DCs. Here, we exposed DCs to chemically inactivated HIV-1 preparations for 24h before co-culture with the specific CD4^+^ T cell clones. Therefore, we added HIV-C-matured DCs to the CD4^+^ T cell clones and this was also demonstrated by Tjomsland et al. [53], who showed a significantly higher stimulation of HIV-specific CD4^+^ T cell clones when using mature, HIV-C-exposed DCs for activation. The higher activation of CD4^+^ and CD8^+^ T cells might be at least partially due to higher co-stimulatory capacity of HIV-C-DCs and induction of MIG, IP10 and IP9. To study the antiviral potential of HIV- or HIV-C-loaded DCs, co-cultures of HIV-exposed BDCA1^+^ DCs and autologous T cells were monitored for infection. These experiments revealed that HIV-C-DCs were prime suppressors of HIV-1 infection, emphasizing the significantly higher antiviral activity induced in DCs by complement-opsonization of HIV. We recently illustrated that antibody opsonization of HIV-1 attenuates the capacity of DCs to stimulate specific CTL responses compared to HIV-C-DCs [32]. Therefore, the innate mechanism initiated in DCs by sole complement opsonization of HIV-1 (in the absence of Ab coating) during the acute phase of infection might be down-modulated after appearance of specific antibodies and interactions via Fc receptors on DCs, a scenario that needs further investigation. Additionally, we found a higher complement and IgG deposition on virus particles using plasma samples from HIV-1-positive individuals for opsonization compared to healthy controls [32]. As demonstrated, complement coating of HIV-1 strongly influences intracellular fates or routes of the virus in DCs compared to non-opsonized HIV-1, thereby profoundly affecting DC infection and activation. Since *in vivo* most viruses will be opsonized by complement immediately upon entry via mucosal surfaces or already in seminal fluid, the dogma of non/low-level infection of DCs by HIV associated with a weak T cell activation should be revisited. In summary, our data provide novel immunologic and mechanistic insights into processes during acute HIV infection, where the host is able to cope with the virus due to efficient cellular and humoral immune responses. These findings might be exploited for future therapeutic options to improve antiviral immune responses via triggering not yet considered host mechanisms, i.e. complement receptor signaling on DCs.

## Materials and Methods

### Ethics statement

Written informed consent was obtained from all participating blood donors by the Central Institute for Blood Transfusion & Immunological Department, Innsbruck, Austria. The use of anonymized leftover specimens for scientific purposes was approved by the Ethics Committee of the Medical University of Innsbruck.

### Monocyte isolation

Monocytes were isolated from the blood of normal healthy donors by using CD14 BD IMAG Beads (Becton-Dickinson), according the manufacturer’s instructions. DCs were generated and analyzed as described [24, 32]. For some experiments, BDCA1^+^ DCs were isolated directly from blood collection bags using the human CD1c (BDCA-1)^+^ DC Isolation Kit (Miltenyi) according to the manufacturer´s instructions.

### Plasmids

The infectious HIV-1 GFP WT proviral clone contains EGFP in the 5′-region of nef and a polypurine track 3′ of EGFP followed by the remaining 3′-LTR sequence [19]. The Vpx expression construct pcDNA3.1Vpx SIVmac239-Myc and VSV-G (pMD.G) were used to obtain Vpx-carrying or VSV-G pseudotyped HIV virus preparations. The infectious HIV-2 GFP WT proviral clone, based on pROD9-ΔEnv-GFP from M. Emerman, was generated by reintroduction of Rod9 Env and virus production was performed as described [19]. Env-deleted (HIVΔenv) HIV-1_BRU_ virus was used for single cycle infection experiments.

### Virus production

Sucrose cushion—purified HIV virus stocks were produced as previously described [56]. HIV-1 and HIV-2 proviral clones were produced by transfection into HEK293T cells. Also, HIV-1*GFP virus stocks, carrying virion-packaged Vpx, were produced by co-transfection of HEK293T cells of the proviral HIV-1*GFP DNA and the indicated Vpx expression constructs [57]. Vesicular Stomatitis Virus type G (VSV-G) pseudotyped viruses (HIV-1, HIV-2) were generated by cotransfection of HEK 293 T cells with the corresponding provirus and the VSV-G expression plasmid pMD.G. R5-tropic HIV-1 (BaL, 92UG037, 92BR030) was propagated in IL-2/PHA-L-stimulated PBMCs.

### Opsonization of HIV-1

To mimic the opsonization process *in vitro*, HIV-1 was incubated for 1h at 37°C with human complement (C) serum (Quidel) in a 1:10 dilution. As negative control the virus was incubated under the same conditions in commercially available C3-deficient serum (Sigma). Subsequent to opsonization, the virus was thoroughly washed to remove unbound components, pelleted by ultracentrifugation (20000 rpm/90 min/4°C) and re-suspended in 200μl RPMI medium without supplements. The opsonization pattern was determined by virus capture assay (VCA) as described [7] using anti-human C3c- (recognizing C3b, iC3b), C3d-, IgG- or mouse IgG Abs as controls for background binding. The coated VCA plates were incubated overnight with the differentially opsonized virus preparations (2.5 ng p24/well) at 4°C, washed 5 times and bound virus was lysed and transferred to a pre-coated p24 ELISA plate [58]. A representative VCA is depicted in S5 Fig.

### DC infection

Day 5 iDCs (1x10^5^ cells/100μl) were infected in triplicates with 25 ng p24/ml differentially opsonized HIV-1 as described [7, 9]. Briefly, to confirm the detection of productive infection of DCs and not cell-associated HIV-1, we thoroughly washed the cells after 3h incubation with differentially opsonized HIV-1 preparations and cultured the cells at 37°C/5% CO_2_. By ELISA we measured the p24 concentrations of the supernatants following spinning down the plate on several days post infection (S1A Fig). Enhancement of DC infection by C-opsonization was verified 5 times in different donor cells and using different HIV-1 isolates.

### Blam-Vpr assay

Viral fusion was analyzed as described [29]. Briefly, day 5 DCs were plated into a 96-well plate in triplicates (1.5x10^5^ cells/100μl) in RPMI in presence of 10mM Hepes (Life Technologies) and 2mg/ml DEAE-Dextran (Sigma-Aldrich). Cells were exposed to the indicated concentrations of non-opsonized (HIV) or complement-opsonized (HIV-C) HIV-1 containing Blam-Vpr. After 3 hours, cells were washed 2 times in CO_2_-independent medium (Life Technologies), re-suspended in CO_2_-independent medium containing 10% FCS and loaded with the CCF2-AM substrate solution (LiveBLAzer FRET-B/G Loading Kit with CCF2-AM, Life Technologies). After 2h incubation at room temperature (dark) cells were washed 2 times in CO_2_-independent medium, fixed in 4% paraformaldehyde for 30 min and respective wells were pooled for flow cytometric analysis.

### Confocal microscopy

To track intracellular HIV localization, iDCs were exposed to GFP-tagged HIV-1 (100 ng p24/ml). DCs were labeled in addition using Draq5 (nucleus, Molecular Probes), Actistain phalloidin (Cytoskeleton), or AlexaFluor 555-labeled IRF3 (Antibodies Online). Confocal microscopy was performed on an LSM 510 microscope, x40OIL (Zeiss) and quantifications of colocalization were done using the Metamorph software (Universal Imaging) on an average of 35–40 cells for each condition [59].

### Immuno-blot analyses of phosphorylated proteins

Samples were lysed 25 min at 4°C in ice-cold 1% Triton-X100 buffer containing protease and phosphatase inhibitors (Sigma-Aldrich). The protein content was determined by Bradford. Cell lysates were separated on 12% SDS-PAGE gels and transferred to PVDF membranes. Membranes were incubated with anti-human phospho-Thr592 SAMHD1 Ab (F. Diaz-Griffero), SAMHD1, NFκB, P-STAT1, ERK1/2 or tubulin Abs (all from Cell Signaling Technology). For every donor, immunoblot quantification was performed using ImageJ and a loading control to normalize the values (ERK1/2, tubulin). Quantifications were performed using values from at least 3 different donors.

### Relative quantification by real-time RT-PCR

mRNA expression of IFNB1 was analyzed by real-time RT-PCR using gene-specific primer/probe pairs (Life Technologies). A GAPDH (human)-PCR using specific primer/probe pairs (Life Technologies) served as internal control to quantify the relative gene expression of target genes. The iScript One-Step RT-PCR Kit for Probes (BioRad) was used for target amplification and runs were performed on the CFX96 real-time detection system (BioRad). The cycling conditions were as follows: 10 min@50°C (RT), 3 min@95°C, 40 cycles: 5 sec@95°C, 15 sec@60°C. The results were analyzed using the gene expression software of the cycler (CFX Manager Software, ΔΔCt method) [60]. Primers and method for HIV-1 dynamics was performed as described [7]. Briefly, integrated HIV-1 levels were determined by a nested PCR comprising an *Alu-*HIV-1 PCR as first step, followed by a second PCR the first cDNA products and R/U5 primers and probe set. Thermal cycling conditions for R/U5 and full-length HIV-DNA consisted of 95°C@10 min and 40 cycles of 15 s@95°C and 30 s@60°C. The *Alu-*HIV-1 PCR was performed at 95°C for 10 min followed by 22 cycles of 30 s at 95°C, 30 s at 66°C, 10 min at 70°C, and a final extension step of 10 min at 72°C. All real-time PCR runs were performed on the CFX96 real-time detection system (Bio-Rad).

### Multicolor FACS analyses

Multicolor FACS analyses of HIV-exposed DCs using anti-human CD83, CD86, CCR7, CD40, HLA-ABC, HLA-DR (all BD Biosciences) were performed as described [32] on a FACS Verse flow cytometer (BD Biosciences). Data were analyzed using FlowLogic Software (Affymetrix).

### HIV-specific CD4^+^ T cell clones, CTLs and Elispot analyses

HIV-specific CD4^+^ T cell clones were generated by *in vitro* priming as described by Moris et al. (54). SL9 clone 2, an HIV-specific CD8^+^ CTL clone, was derived from an HIV-infected patient and recognizes the well-characterized immune-dominant epitope of Gag p17 SLYNTVATL (SL9) presented by HLA-A*020 [46, 61]. DCs were co-cultured overnight with the T cell clones (2500 to 10000 clones/well). As positive controls, DCs were incubated with 1μg/ml of cognate peptide before washing and addition of the HIV-specific CD4^+^ T cell or CTL clones overnight. IFN-γ production was monitored in an Elispot assay as described (61). All Abs used for the IFN-γ Elispot were from Mabtech.

### Statistical analysis

Differences were analyzed by the GraphPad prism software using the unpaired student’s t-test (2-tailed) or one-way ANOVA with Bonferroni posttest for multiple comparisons depending on the analyses.

## Supporting Information

S1 Fig (A) Higher DC infection with C-opsonized HIV. Kinetics of DC infection with HIV, HIV-C, HIV*Vpx or HIV*Vpx-C revealed that over time, HIV-C (red) caused a high productive infection of DCs comparable to that of HIV*Vpx DCs (green). Complement-opsonization furthermore enhanced the productive infection of Vpx-carrying HIV (yellow). As expected, non-opsonized HIV caused a low level infection in DCs (blue). One representative infection assay performed in triplicates (out of 5) is depicted. *(B)* Additionally, BDCA1^+^ DCs directly isolated from blood of three different donors were infected using non- or complement-opsonized HIV-1 (25 ng p24/ml) and productive infection was monitored on several dpi. A summary of the three donors of day 12 pi illustrates the significant complement-mediated enhancement of DC infection also in BDCA1^+^ DCs compared to HIV-BDCA1^+^ DCs (p = 0.0004). Non-infected BDCA1^+^ DCs served as negative controls. Statistical differences between HIV- and HIV-C DCs were evaluated using an unpaired Student´s t test. *(C)* Also in FACS analyses two times as much infected DCs were measured with HIV-C, HIV*Vpx and HIV*Vpx-C compared to HIV-exposed DCs. We gated on live, CD11c^high^ and GFP^+^ cells. FACS analyses were repeated in 3 independent experiments.
(TIF)

S2 Fig (A) Similar entry of differentially opsonized HIV-1 in DCs. DCs (1,5x10^5^/100μl) were exposed to the indicated doses of non- (HIV) or complement-opsonized (HIV-C) HIV-1 bearing the chimeric protein β–lactamase-Vpr (Blam-Vpr). After 3h at 37°C, cells were washed 2 times and CCF2-AM dye was loaded. After 2h incubation at room temperature cells were washed 2 times, fixed in 4% paraformaldehyde for 30 min and viral access to the cytoplasm was measured by flow cytometry using the ability of β–lactamase to cleave the cytoplasmic CCF2-AM substrate. The experiment was repeated using cells from three different donors. *(B) More effective transcription of HIV-C in DCs*. When characterizing relative expression levels of full-length (FL) and integrated HIV-DNA in DCs, significantly higher expression levels of FL HIV DNA (p = 0.0044) and about 100-fold higher incorporation of the viral into the host genome were detected in HIV-C DCs compared to HIV DCs. These analyses were repeated using cells from four donors.
(TIF)

S3 Fig No DC maturation upon incubation with C-opsonized beads. To rule out that C opsonization alone accounts for DC maturation and activation, DCs were incubated with non- and C-opsonized Beads. Both Beads and Beads-C caused a slight maturation comparable to background signals by iDCs (6.2% vs. 4.1% vs. 2.5% CD83^+^ DCs). LPS-stimulated DCs were used as positive controls (88.9% CD83^+^ DCs). This experiments war repeated thrice.
(TIF)

S4 Fig HIV-C-DCs show an increased expression of type I IFN-associated and T cell-stimulatory genes compared to HIV-DCs. *(A)* mRNA expression of IFNB1 from an additional donor is illustrated (description see Fig 4B). *(B)* DCs were infected with non- or C-opsonized HIV-1 (HIV, HIV-C) at an MOI 0.5 and 24h after infection total RNA was isolated, labeled and analyzed using the Agilent High-Resolution Microarray Scanner. Among many other differentially regulated genes, genes associated with Type I IFN response and T cell stimulatory capacity were stimulated to greater levels in HIV-C-DCs compared to HIV-DCs. Microarrays were performed using DCs from 5 different donors treated with LPS or the R5-tropic BaL or primary isolate 92UG037. Treated DCs were normalized to untreated controls (iDCs). Heat-maps of LPS-, HIV- or HIV-C-treated DCs normalized to iDCs are depicted in yellow-red for a better discrimination of the ISG gene expression between the samples and additionally HIV was compared versus HIV-C, which is depicted in grey-black.
(TIF)

S5 Fig Characterization of the viral surface after opsonization. C3c-, C3d- and IgG- deposition on the HIV surface opsonized with medium/C3-deficient serum (HIV) or human complement serum (Quidel) (HIV-C) was characterized by VCA as described in the Methods section. While HIV did not bind to any of the coated Abs (human C3c, C3d, IgG), a strong binding of HIV-C to C3c and C3d was observed and only background binding to human IgG. Coating the plate using a mouse IgG Ab served as negative control for background binding of the virus preparations. VCA is routinely performed after opsonization of HIV and a representative graph is depicted.
(TIF)

S1 Text Supporting methods. (DOCX)
