# Supplementary figures and images for: Complement-Opsonized HIV-1 Overcomes Restriction in Dendritic Cells
Source: PLoS Pathog. 2015 Jun 29;11(6):e1005005. doi: 10.1371/journal.ppat.1005005 (PMC4485899; doi:10.1371/journal.ppat.1005005)

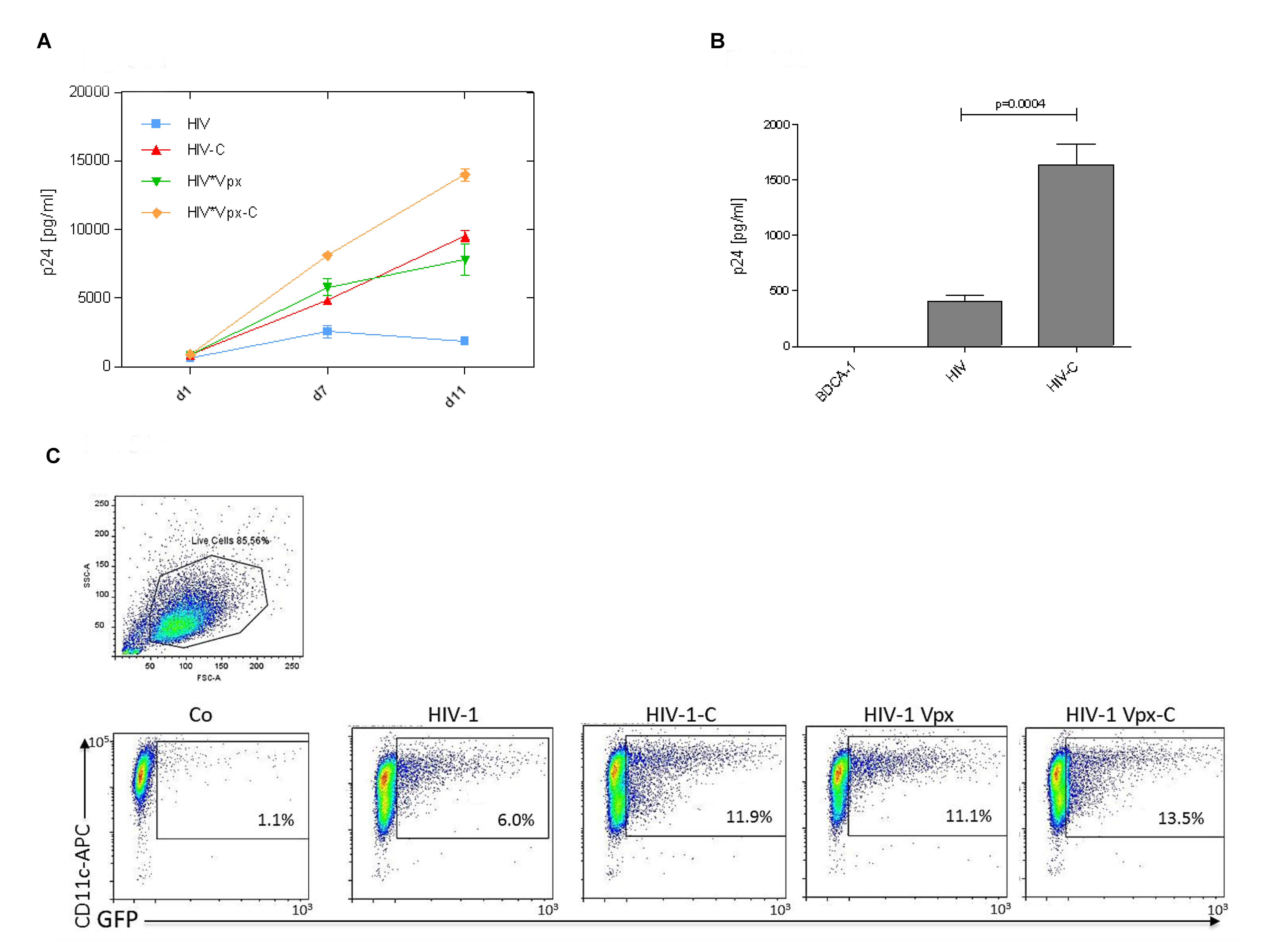

Supplement: S1 Fig — Kinetics of DC infection with HIV, HIV-C, HIV*Vpx or HIV*Vpx-C revealed that over time, HIV-C (red) caused a high productive infection of DCs comparable to that of HIV*Vpx DCs (green). Complement-opsonization furthermore enhanced the productive infection of Vpx-carrying HIV (yellow). As expected, non-opsonized HIV caused a low level infection in DCs (blue). One representative infection assay performed in triplicates (out of 5) is depicted. (B) Additionally, BDCA1+ DCs directly isolated from blood of three different donors were infected using non- or complement-opsonized HIV-1 (25 ng p24/ml) and productive infection was monitored on several dpi. A summary of the three donors of day 12 pi illustrates the significant complement-mediated enhancement of DC infection also in BDCA1+ DCs compared to HIV-BDCA1+ DCs (p = 0.0004). Non-infected BDCA1+ DCs served as negative controls. Statistical differences between HIV- and HIV-C DCs were evaluated using an unpaired Student´s t test. (C) Also in FACS analyses two times as much infected DCs were measured with HIV-C, HIV*Vpx and HIV*Vpx-C compared to HIV-exposed DCs. We gated on live, CD11chigh and GFP+ cells. FACS analyses were repeated in 3 independent experiments. (TIF) [file ppat.1005005.s001.tif]

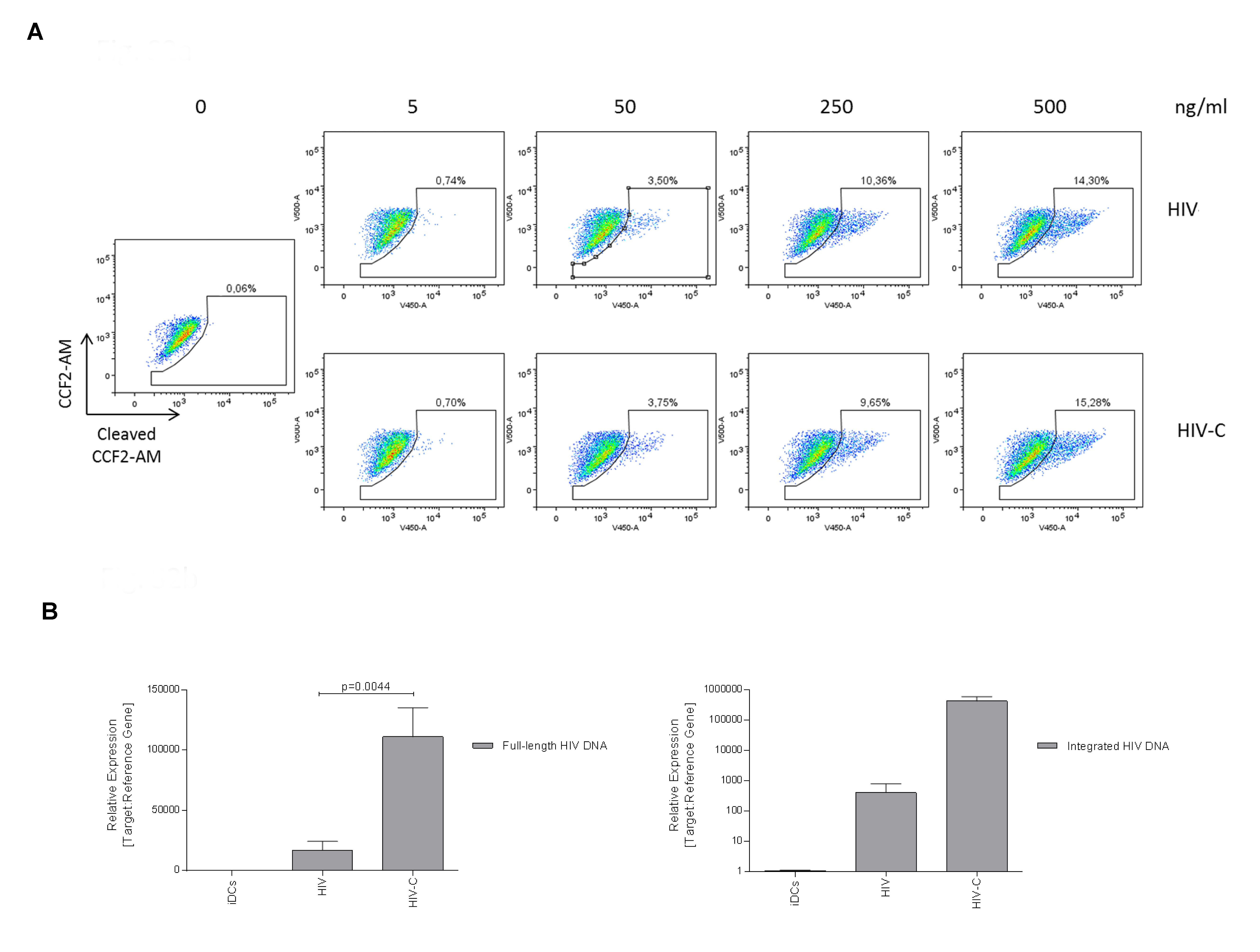

Supplement: S2 Fig — DCs (1,5x105/100μl) were exposed to the indicated doses of non- (HIV) or complement-opsonized (HIV-C) HIV-1 bearing the chimeric protein β–lactamase-Vpr (Blam-Vpr). After 3h at 37°C, cells were washed 2 times and CCF2-AM dye was loaded. After 2h incubation at room temperature cells were washed 2 times, fixed in 4% paraformaldehyde for 30 min and viral access to the cytoplasm was measured by flow cytometry using the ability of β–lactamase to cleave the cytoplasmic CCF2-AM substrate. The experiment was repeated using cells from three different donors. (B) More effective transcription of HIV-C in DCs. When characterizing relative expression levels of full-length (FL) and integrated HIV-DNA in DCs, significantly higher expression levels of FL HIV DNA (p = 0.0044) and about 100-fold higher incorporation of the viral into the host genome were detected in HIV-C DCs compared to HIV DCs. These analyses were repeated using cells from four donors. (TIF) [file ppat.1005005.s002.tif]

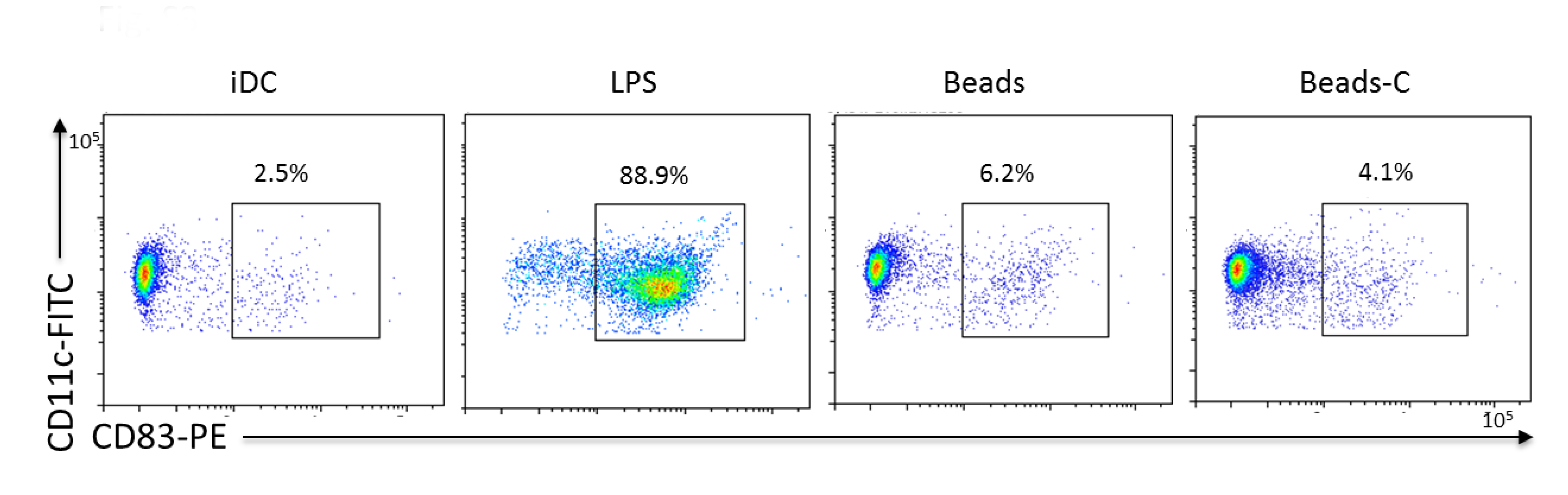

Supplement: S3 Fig — To rule out that C opsonization alone accounts for DC maturation and activation, DCs were incubated with non- and C-opsonized Beads. Both Beads and Beads-C caused a slight maturation comparable to background signals by iDCs (6.2% vs. 4.1% vs. 2.5% CD83+ DCs). LPS-stimulated DCs were used as positive controls (88.9% CD83+ DCs). This experiments war repeated thrice. (TIF) [file ppat.1005005.s003.tif]

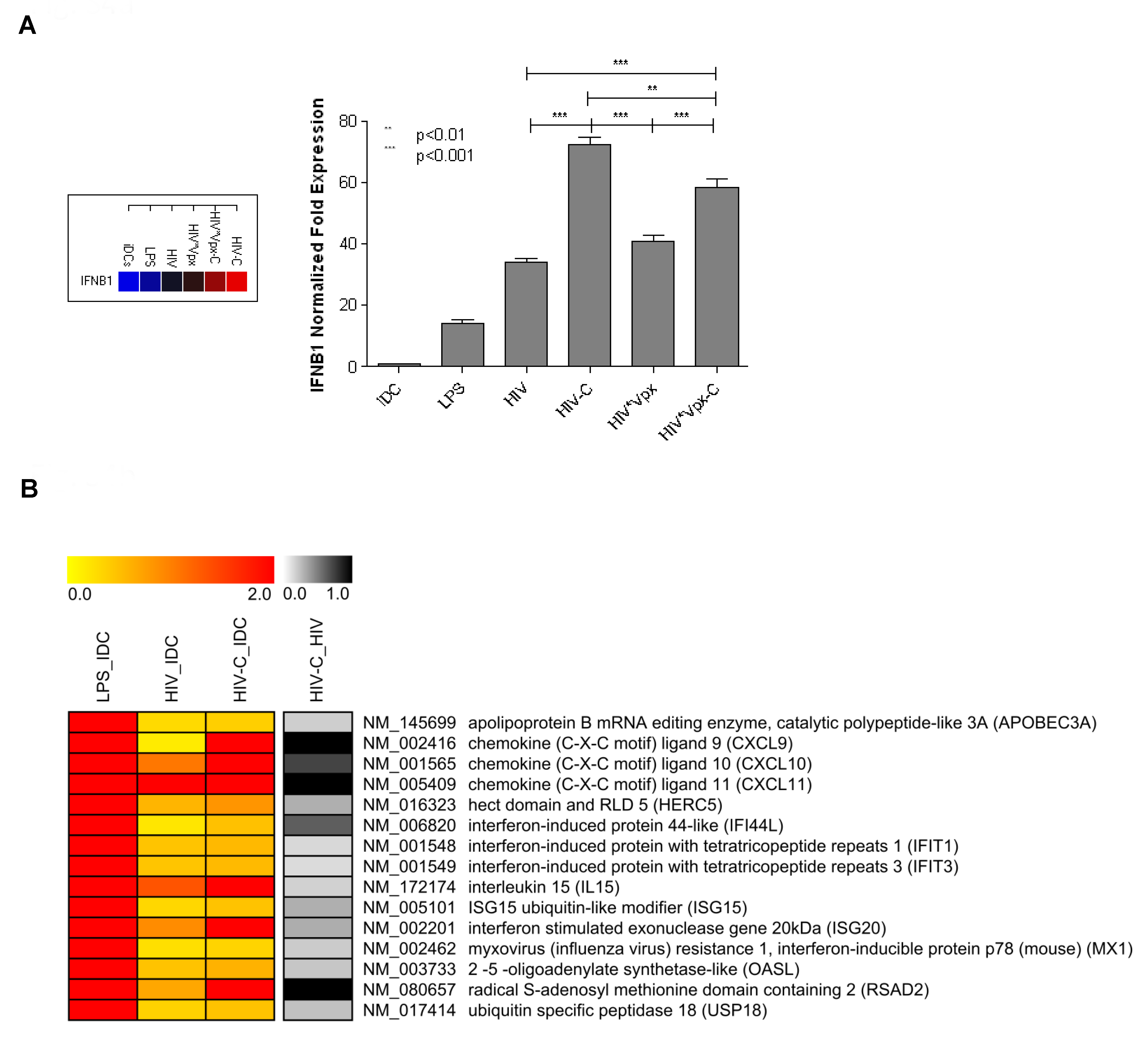

Supplement: S4 Fig — (A) mRNA expression of IFNB1 from an additional donor is illustrated (description see Fig 4B). (B) DCs were infected with non- or C-opsonized HIV-1 (HIV, HIV-C) at an MOI 0.5 and 24h after infection total RNA was isolated, labeled and analyzed using the Agilent High-Resolution Microarray Scanner. Among many other differentially regulated genes, genes associated with Type I IFN response and T cell stimulatory capacity were stimulated to greater levels in HIV-C-DCs compared to HIV-DCs. Microarrays were performed using DCs from 5 different donors treated with LPS or the R5-tropic BaL or primary isolate 92UG037. Treated DCs were normalized to untreated controls (iDCs). Heat-maps of LPS-, HIV- or HIV-C-treated DCs normalized to iDCs are depicted in yellow-red for a better discrimination of the ISG gene expression between the samples and additionally HIV was compared versus HIV-C, which is depicted in grey-black. (TIF) [file ppat.1005005.s004.tif]

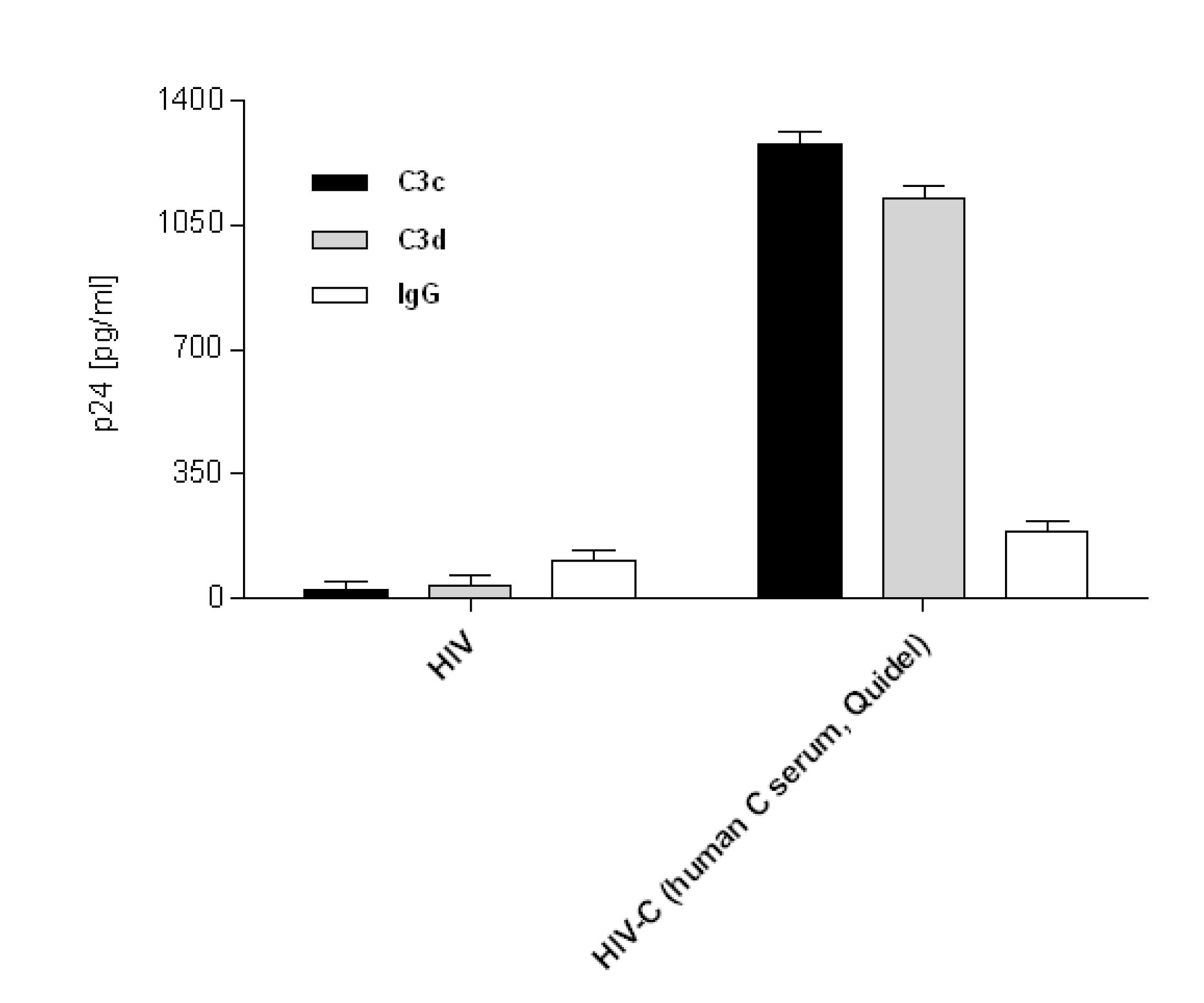

Supplement: S5 Fig — C3c-, C3d- and IgG- deposition on the HIV surface opsonized with medium/C3-deficient serum (HIV) or human complement serum (Quidel) (HIV-C) was characterized by VCA as described in the Methods section. While HIV did not bind to any of the coated Abs (human C3c, C3d, IgG), a strong binding of HIV-C to C3c and C3d was observed and only background binding to human IgG. Coating the plate using a mouse IgG Ab served as negative control for background binding of the virus preparations. VCA is routinely performed after opsonization of HIV and a representative graph is depicted. (TIF) [file ppat.1005005.s005.tif]
