# Supplementary material for: Complement-Opsonized HIV-1 Overcomes Restriction in Dendritic Cells
Source: PLoS Pathog. 2015 Jun 29;11(6):e1005005. doi: 10.1371/journal.ppat.1005005 (PMC4485899; doi:10.1371/journal.ppat.1005005)
Supplement: S1 Text — (DOCX) [file ppat.1005005.s006.docx]

**Supporting Information Methods**

***Microarray analyses***

For microarray analyses, day 5 iDCs (0.5x10^6^ cells) were exposed to LPS (100 ng/ml, Sigma-Aldrich, St Louis, MO), non- or complement-opsonized HIV-1 preparations (300 ng p24/ml, MOI~0.5), or left untreated for 24h. Total RNA was extracted with the RNeasy Plus Mini Kit (Qiagen, Germany). The RNA quality was tested using the 2100 Bioanalyzer (Agilent, CA, USA) and fluorescent cRNA was generated by Agilent´s Quick Amp Labeling Kit for 2-color processing, according to the manufacturer´s instructions using 500 ng sample input RNA. The labeled/amplified cRNA was purified (Qiagen, Germany) and quantified using NanoDrop (Thermo Scientific, MA, USA). Yield and specific activity (SA) of each reaction were determined by 2 equations (yield: (Concentration of cRNA) * 30 μL (elution volume) / 1000 = μg of cRNA; SA: (Concentration of Cy3 or Cy5) / (Concentration of cRNA) * 1000 = pmol Cy3 per μg cRNA). If the yield was >825ng and the SA >8.0 pmol Cy3 or Cy5 per µg cRNA, hybridization was performed according to Agilent´s instructions using the labeled cRNA and the Agilent Gene Expression Hybridization Kit. Image acquisition and analyses were performed using the Agilent High-Resolution Microarray Scanner following the manufacturer´s protocols. Information about probe features was extracted from microarray scan data by Agilent Feature Extraction Software Extraction (FE) 9.5.3 according to GE2-v4_95_Feb07 default protocol. Local background subtracted features from Agilent FE software were standardized to the Reference sample (RNA) (Cy3/Cy5), log2-transformed, and shifted to the 50% percentile (median normalization within array). The scale factor was normalized across arrays. Control features and empty spots were appropriately filtered. All experimental parameters, protocols, raw and transformed data were submitted to a public repository (ArrayExpress, E-MEXP-3706). Differentially expressed genes were identified using moderated t-test (R/Bioconductor package ‘limma’) in a paired analysis for each DC preparation. P-values were adjusted for multiple hypothesis testing based on the false discovery rate by the Benjamini-Hochberg method. Genes were considered differentially expressed in one of the treated conditions compared to iDCs if adjusted p-value<0.05 and fold-change ≥ 2.
